# Transcriptome of *Dickeya dadantii* Infecting *Acyrthosiphon pisum* Reveals a Strong Defense against Antimicrobial Peptides

**DOI:** 10.1371/journal.pone.0054118

**Published:** 2013-01-14

**Authors:** Denis Costechareyre, Jean-François Chich, Jean-Marc Strub, Yvan Rahbé, Guy Condemine

**Affiliations:** 1 Université de Lyon, Lyon, France; 2 Université Lyon 1, Villeurbanne, France; 3 CNRS UMR5240, Microbiologie Adaptation et Pathogénie, Villeurbanne, France; 4 INSA-Lyon, Villeurbanne, France; 5 INRA UMR1131, Santé de la Vigne et Qualité du Vin, Colmar, France; 6 CNRS UMR7178, Laboratoire de Spectrométrie de Masse BioOrganique, IPHC-DSA- Université de Strasbourg, Strasbourg, France; 7 INRA UMR203, Biologie Fonctionelle Insectes et Interaction, Villeurbanne, France; University of Wisconsin-Milwaukee, United States of America

## Abstract

The plant pathogenic bacterium *Dickeya dadantii* has recently been shown to be able to kill the aphid *Acyrthosiphon pisum*. While the factors required to cause plant disease are now well characterized, those required for insect pathogeny remain mostly unknown. To identify these factors, we analyzed the transcriptome of the bacteria isolated from infected aphids. More than 150 genes were upregulated and 300 downregulated more than 5-fold at 3 days post infection. No homologue to known toxin genes could be identified in the upregulated genes. The upregulated genes reflect the response of the bacteria to the conditions encountered inside aphids. While only a few genes involved in the response to oxidative stress were induced, a strong defense against antimicrobial peptides (AMP) was induced. Expression of a great number of efflux proteins and transporters was increased. Besides the genes involved in LPS modification by addition of 4-aminoarabinose (the *arnBCADTEF* operon) and phosphoethanolamine (*pmrC*, *eptB*) usually induced in Gram negative bacteria in response to AMPs, *dltBAC* and *pbpG* genes, which confer Gram positive bacteria resistance to AMPs by adding alanine to teichoic acids, were also induced. Both types of modification confer *D. dadantii* resistance to the AMP polymyxin. *A. pisum* harbors symbiotic bacteria and it is thought that it has a very limited immune system to maintain these populations and do not synthesize AMPs. The *arnB* mutant was less pathogenic to *A. pisum*, which suggests that, in contrast to what has been supposed, aphids do synthesize AMP.

## Introduction

*Dickeya dadantii* are plant pathogenic enterobacteria that provoke the soft rot disease in a wide range of plants. The factors required for its pathogeny are numerous, involving principally the production and the secretion of enzymes responsible for the degradation of plant cell wall components [1], [2]. Regulation of their expression has been studied in detail. The regulators of the virulence factors identified are either specific to *D. dadantii* and other plant pathogenic enterobacteria, such as KdgR, PecS and PecT, or are identical to those that can be found in animal and human pathogenic enterobacteria such as *Escherichia coli* and *Salmonella enterica* [3]. These regulators include H-NS, CRP, Fur, GacS-GacA and PhoP-PhoQ [4], [5], [6], [7]. The PhoP-PhoQ two component regulatory system controls virulence and Mg^2+^ homeostasis in many bacterial species. Its role has been well studied in *S. enterica* serovar typhimurium. In these bacteria, this system is involved in the regulation of Mg^2+^ uptake systems, survival in macrophages and resistance to antimicrobial peptides (AMP) [8]. This resistance occurs through the modification of LPS. Several enzymes, encoded by *pagP*, *pagO*, *pmrC*, *pmrG*, *lpxO*, *pmrHFIJKLM*, modify LPS, mostly by adding or modifying palmitate, phosphoethanolamine or 4-aminoarabinose to mask negative charges that allow interaction with cationic AMP [9]. Activation of these genes occurs either directly by PhoP-PhoQ or by PmrA-PmrB, a two component system that can be activated by PhoP through PmrD, a protein stabilizing PmrA in a phosphorylated state. Thus, the genes controlled by PmrA-B are induced both by the signals activating PmrB (macrophage phagosome, high Fe^3+^, low pH) and those activating PhoQ (low Mg^2+^, antimicrobial peptides, macrophage phagosome, low pH) [9]. However, the genes regulated by PhoP and the signal sensed by PhoQ may vary from one bacterium to the other [10]. For exemple, *Edwarsiella tarda* PhoQ is also able to sense temperature, *Pseudomonas aeruginosa* PhoQ does not respond to AMP, and *Sodalis glossinidius* PhoQ responds neither to Mg^2+^ nor to AMP [11], [12], [13]. An analysis of the transcriptome of a *D. dadantii phoQ* mutant showed that many genes are controlled by this regulator. The increased expression of ferric uptake systems and the decreased expression of pectate lyase genes let suppose that *phoQ* could control the bacterial virulence [14]. Llama-Palacios *et al*. [7] showed that the *D. dadantii phoP* and *phoQ* mutants have a reduced virulence and an increased sensitivity to the plant AMP thionin. A transcriptomic analysis of the response of *D. dadantii* to this AMP found 36 overexpressed genes. These induced genes are involved in regulation, transport and modification of the bacterial membrane [15].

*D. dadantii* is not only a plant pathogen but it can also infect animals. Presence of four insecticidal toxin-like genes in its genome, *cytABCD* [16], led to check for a possible pathogenicity towards different insects. It was found able to kill the pea aphid *Acyrtosiphon pisum* but not other insects tested [17]. Ingestion of as few as 100 bacteria can kill the aphid in 3–4 days. Bacteria cross the gut and after one day they are present in the gut in high number but also in the fat bodies and in the embryos. Their number increases of about one log each day and when it exceedes 10^7^ bacteria/insect, the insect dies [18]. A mutant devoid of Cyt toxin genes is still pathogenic but kills the aphid more slowly, in 5 to 6 days. *cyt* genes are controlled by regulators of plant pathogenicity: they are activated by PecS and repressed by H-NS and VfmE. Mutants in other regulators, GacA, OmpR and PhoP, that do not control the *cyt* genes, have a reduced insect virulence. This let suppose that other factors are required for a full virulence to aphids [19]. However, no other known insect toxin gene was detected in the genome of *D. dadantii*.

Very few data are available on the expression pattern of genes of bacteria infecting insects. A transcriptome analysis listed the *Yersinia pestis* genes induced in flea, but *Y. pestis* colonizes only the flea gut [20]. Thus, this study gives a limited pattern of the bacterial response to conditions encountered in insects. A SCOTS analysis of *Photorhabdus temperata* and *Xenorhabdus koppenhoeferi* genes expressed in *Rhizotrogus majalis* identified genes mostly involved in virulence, stress response and metabolism [21]. A Transposon Site Hybridization assay identified *Francisella novicida* genes required to infect *Drosophila melanogaster* [22]. Moreover, in all these studies, infection was done by injection, which is not the most likely path to infection. To identify new factors involved in the virulence of *D. dadantii* towards *A. pisum*, we analyzed the transcriptome of *D. dadantii* infecting the insect. We show here that genes involved in resistance to AMP, efflux and motility are among the most induced genes while genes involved in plant pathogeny are repressed. Mutation of AMP resistance genes decreased bacterial virulence in insects which is a strong indication that, in contrast with current knowledge, aphids are producing this kind of molecules.

## Results

### Identification of *D. Dadantii* Genes with Modified Expression in *A. Pisum*

In order to identify genes that are important for growth inside the pea aphid and that may be required for virulence, we used CDS pangenomic microarrays to compare the transcriptome of *D. dadantii* grown in AP3 medium with that of the bacteria isolated from *A. pisum* three days after infection. AP3 medium, which contains a high concentration of sucrose, may mimic the phloemic sap on which aphids feed. Day three post infection was chosen as a stage where infection is well established. At this stage, aphids may contain up to 10^6^ bacteria which are present in all the insect organs. For each condition, three independent cultures or infections were performed and the total bacterial RNA was extracted, treated and subsequently hybridized to separate arrays. Major modifications of the transcription pattern were observed since 164 genes were upregulated more than 5-fold and 328 genes were downregulated more than 5-fold with *P* values adjusted for multiple testing (FDR) of <0.002. The whole-genome GSEA (Gene Set Enrichment Analysis) analysis is reported in , and shows that the most affected bacterial gene classes by the insect infection process were membrane associated genes (GO *cell compartment*), both in up or down-regulated classes, RNA and ribosomal machinery genes, as well as many enzyme and sugar transporters (GO *molecular function*) and finally additional classes such as protein secretion, several biosynthetic processes, cell redox homeostasis and many central metabolic processes (GO *biological processes*). A list of selected induced or repressed genes that are discussed in the text is given in Table 1 (for the full list, see Table S2). The gene expression pattern obtained by microarray data results was confirmed by qRT-PCR on nine genes. Six up regulated (*arnB*, *sotA*, *sotB*, *pmrC*, GenID 19611 and GenID 15786) and three down regulated genes (*pelE*, *kdgM* and *kdgN*) were tested and gave a strong correlation with microarray results (Fig. S1).

**Table 1:** A subset of *D. dadantii* genes whose expression varies in aphid.

Gene product category and ID	Gene name	Fold change^a^	*P*-value	Description
**Upregulated genes**				
AMP response				
15458	*pmrC*	90.8	<0.002	Phosphoethanolamine transferase
16248	*arnB*	64.0	<0.002	UDP-4-amino-4-deoxy-L-arabinose alpha-ketoglutarate aminotransferase
16247	*arnC*	36.1	<0.002	Undecaprenyl-phosphate 4-amino-4-deoxy-L-arabinose transferase
18941	*pbpG*	12.7	<0.002	D-alanyl-D-alanine carboxypeptidase
19383	*dltB*	32.0	0.02	D-alanyl transfer protein
19382	*dltA*	20.1	0.02	D-alanine-activating enzyme
19381	*dltC*	8.9	0.03	D-alanine carrier protein
16330	*eptB*	6.5	0.02	Phosphoethanolamine transferase
Efflux system, transporter				
15786		68.6	<0.002	Inner membrane component of multidrug resistance system
15787		48.2	<0.002	Membrane fusion component of multidrug resistance system
19611		37.7	<0.002	Transport protein (MFS family)
18887		24.4	<0.002	Transport protein (MFS family)
16087	*pecM*	24.1	<0.002	Transport protein
20022	*sotB*	20.7	<0.002	Transport protein (MFS family)
20031	*sotA*	14.4	<0.002	Transport protein (MFS family)
15661		11.9	<0.002	Drug resistance efflux pump
15662		10.1	<0.002	Drug resistance efflux pump
18175		10.2	0.003	ABC transport system
18174		5.6	0.01	ABC transport system
Motility				
19858		43.4	<0.002	Methyl-accepting chemotaxis protein
19855		30.7	<0.002	Methyl-accepting chemotaxis protein
17672		8.3	<0.002	Methyl-accepting chemotaxis protein
15600		8.1	<0.002	Methyl-accepting chemotaxis protein
17668		7.5	<0.002	Methyl-accepting chemotaxis protein
17665		7.3	<0.002	Methyl-accepting chemotaxis protein
18761	*motA*	14.9	<0.002	Flagellar motor protein
18760	*motB*	10.9	<0.002	Flagellar motor protein
Stress response				
14750	*asr*	26.0	<0.002	Acid shock protein
20273	*narI*	21.8	<0.002	Nitrate reductase. Anaerobiosis
18171	*iscR*	11.9	<0.002	FeS cluster assembly, transcription factor
15559	*rsxA*	10.1	<0.002	SoxR reducing complex
15558	*rsxB*	9.2	<0.002	SoxR reducing complex
17401	*nirB*	5,4	<0.002	Nitrite reductase. Anaerobiosis
Regulator				
15788		61.2	<0.002	Transcription regulator
16073	*vfmE*	9.6	0.007	Virulence regulator
**Downregulated genes**				
Pectin catabolism				
19629	*kdgM*	−92.2	<0.002	Oligogalacturonate porin
15523	*kdgN*	−55.0	<0.002	Oligogalacturonate porin
19632	*paeX*	−47.0	<0.002	Pectin acetylesterase
19646	*pelE*	−30.9	<0.002	Pectate lyase E
18695	*exuT*	−21.4	<0.002	Galacturonate transporter
18698	*uxaB*	−18.1	<0.002	Galacturonate catabolism
20789	*pehN*	−9.5	<0.002	Polygalacturonase N
19699	*uxaA*	−8.7	<0.002	Galacturonate catabolism
19960	*kdgA*	−6.7	<0.002	Galacturonate catabolism
18229	*outC*	−6.5	<0.002	Pectate lyase secretion
47127	*kduD*	−5.4	0.002	Pectin catabolism
Galactan catabolism				
18200	*ganL*	−53.2	<0.002	Galactan porin
18192	*ganE*	−41.5	<0.002	Galactan transport
18377	*mglB*	−38.4	<0.002	Galactose transport
18193	*ganF*	−27.8	<0.002	Galactan transport
18195	*ganG*	−24.2	<0.002	Galactan transport
18378	*mglA*	−20.4	<0.002	Galactose transport
18379	*mglC*	−19.0	<0.002	Galactose transport
18196	*ganA*	−17.2	<0.002	Endogalactanase
18198	*ganB*	−7.3	<0.002	Exogalactanase
osmoregulation				
19710	*betI*	−72.5	<0.002	Betaine synthesis
16548	*scrY*	−53.2	<0.002	Sucrose porin
19708	*betA*	−52.2	<0.002	Betaine synthesis
19709	*betB*	−31.5	<0.002	Betaine synthesis
16547	*scrA*	−31.0	<0.002	Sucrose metabolism
19635		−22.0	<0.002	Osmotically induced lipoprotein
Toxin				
16662	*cytD*	−3.5	<0.002	Insecticidal toxin
16663	*cytC*	−2.0	<0.002	Insecticidal toxin
16664	*cytB*	−2.8	<0.002	Insecticidal toxin
16665	*cytA*	−1.6	<0.002	Insecticidal toxin
stress				
16995	*raiA*	−92.6	<0.002	Cold shock protein
16379	*uspA*	−25.8	<0.002	Universal stress protein
16377	*uspB*	−9.5	<0.002	Universal stress protein
19170	*cpxR*	−12.4	<0.002	Transcriptional regulator
19171	*cpxP*	−12.3	<0.002	Stress resistance protein

a Positive values represent genes upregulated in aphids, whereas negative values represent genes downregulated.

### Plant Virulence Genes are Repressed in Aphids

Our previous results showed that the regulators controlling *cyt* genes are identical to those regulating plant virulence factors, but that they act in an opposite way [19]. The microarray analysis confirms these observations on a wider basis since many genes involved in pectin degradation, which are induced in plant infection, are repressed. The genes of the two oligogalacturonate-specific porins [23], [24] are among the most repressed (*kdgM*, -92-fold change; *kdgN*, −55-fold change). Genes of some secreted enzymes involved in pectin degradation were also down regulated (*pelE*, *paeX*, *pehN*) (Table 1), as were genes involved in the transport and catabolism of pectin breakdown products (*exuT*, *uxaB*, *uxaA*, *kduD*, *kdgA*). The complete catabolic pathway of galactan, another component of plant cell walls, is also strongly repressed (−52-fold change for the gene of the porin GanL). Expression of the Out type II secretion system (T2SS), that secretes mostly pectinases is also reduced (*outC*, −6.5-fold change). In contrast, expression of the second T2SS, Stt, which secretes the pectin lyase PnlH is slightly increased (*sttI*, 5.3-fold change).

### *D. Dadantii* Genes Induced in Aphids

The most induced genes in this transcriptome analysis probably correspond to those which are the most needed to infect and survive in aphids. It is remarkable to note that many of these genes encode exporters (Table 1). GenID 15786-7-8, with 68-, 48- and 64-fold change, respectively, encode a tripartite multidrug resistance system whose best homologues are found in plant pathogenic or plant-associated bacteria (*Pseudomonas* sp., *Xanthomonas* sp.) and also in insect pathogens (*P. entomophila*, *Arsenophonus nasoniae*) or some insect symbionts (*Baumannia cicadellinicola*). This transporter confers to *D. dadantii* resistance to phytoalexins and protamine [25]. Expression of four genes encoding transporters of the MFS family is also induced: GenID 19611 (37-fold change), GenID 18887 (24-fold change), *sotA* (14-fold change) and *sotB* (20-fold change). If the specificity of the two first exporters is not known, SotA and SotB have been shown to be able to export sugar and sugar derivatives that can be toxic to the bacteria [26]. The gene of the efflux protein PecM is induced 24-fold. *pecM* belongs to the *pecS* regulon and it has been proposed that it could efflux indigoidine, a blue pigment synthesized by the product of the *indABC* genes, which also belong to the *pecS* regulon. Surprisingly, the *indABC* genes are only weekly induced and other *pecS*-controlled genes are not induced, suggesting that PecM could be controlled by another regulator and efflux other types of molecules [27]. Induction of all these exporters shows that *D. dadantii* encounters hostile conditions in aphid and has to efflux many noxious compounds.

Expression of motility genes is strongly modified in the aphid. *D. dadantii* possesses 45 methyl-accepting chemotaxis proteins (MCP) which are often organized in clusters. Expression of some of these genes was increased. For example, in the four MCP gene cluster GenID 19858-GenID 19855-GenID 19852-Gen ID19851, expression of the two first increased 43-fold and 30-fold respectively, while that of the two last genes was unmodified (Table 1). Similarly, in the four MCP gene cluster GenID 17665-GenID 17668-GenID 17672-GenID 17674, expression of the three first genes increased 7-fold to 8-fold, while that of the last gene was unmodified. Expression of the genes of two components of the flagellar motor MotA and MotB increased 15-fold and 11-fold, respectively (Table 1). Colonization of the insect body occurs very quickly, within one day after infection [18]. Induction of these MCP and motility proteins could help the bacteria to quickly reach all the part of the insect body.

### Osmotic Regulation of Cyt Toxin Genes

We have shown that the Cyt toxins are required only for infection by ingestion and that they can be detected in the gut but not in other organs, suggesting that they are not produced throughout all the infection process [18]. Moreover, their synthesis is induced in high osmolarity environment. These observations explain why *cyt* gene expression is surprisingly diminished in our results (Table 1). For the reference condition, bacteria were grown in the sucrose rich (20%) AP3 medium which strongly induces *cyt* gene expression. Bacteria collected in the aphids reside mainly outside of the gut, in the fat body or other organs of the insect in which osmolarity is low [18]. This difference of osmolarity between the two conditions could explain why *cyt* genes are globally less expressed in the aphids in our experiment. This is also the reason why some of the most repressed genes in this analysis are those involved in sucrose transport and metabolism such as the sucrose porin gene *scrY* (-53-fold change), or in osmoprotectant synthesis and transport (*betA*, -52-fold change and *proX*, -23-fold change) (Table 1). We thus checked whether the strong induction or repression observed for certain genes could result from an osmotic regulation. *arnB* and *dltB* expression (see below) were repressed only three-fold and 1.3-fold respectively by growth in 10% sucrose (data not shown). *kdgM* and *kdgN* expression is not regulated by osmolarity [23], [24]. Thus, osmoregulation seems to control only a limited subset of the genes induced or repressed in our transcriptomic analysis.

### Response to Antimicrobial Peptides

Some of the most induced genes in this study are those homologous to genes conferring resistance to AMP in other enterobacteria by modifying LPS. A high induction (91-fold change) was observed for *pmrC*, a gene encoding the protein that adds phosphoethanolamine to lipid A (Table 1). *pmrC* is the first gene of an operon containing also *pmrA* and *pmrB*, which encode a two component regulation system that regulates genes in response to AMP in *S. enterica*. *pmrA* and *B* are induced about 3-fold. Proteins involved in the synthesis and addition to LPS of 4-aminoarabinose are encoded by the *arnBCADTEF* operon (also called *pmrHFIJKLM* or *pbgP-E*) which is strongly induced in *D. dadantii* infecting aphids (64-fold change for *arnB*). Modification of LPS by 4-aminoarabinose allows *S. enterica* to resist to AMP and it has been shown that this modification is required for full virulence of *P. luminescens* towards the greater wax moth, *Galleria mellonella* [28]. A last set of genes potentially involved in resistance to AMP can be noticed: *dltD* (or *pbpG*) (12-fold change) and the *dltBAC* operon (32-fold change) (Table 1). Homologues of these genes are found in Gram positive bacteria where their products modify teichoic acids by adding alanyl residues. Teichoic acids are cell wall glycopolymers which are not found in Gram negative bacteria. Their modification by alanylation masks their negative charges and confers to the bacteria resistance to AMPs [29]; [30]. In Gram negative bacteria *dltABC* and *dltD* genes are present in the phytopathogenic bacteria *Dickeya* sp, *Pectobacterium* sp. and in the entomopathogenic bacteria *P. luminescens*. However, the substrate potentially modified in these bacteria remains to be identified.

Induction of genes responsible for the resistance to AMPs in *D. dadantii* infecting aphids is surprising. The genes coding for AMPs usually produced by insects were not identified in the *A. pisum* genome and no AMP synthesized by the pea aphid has been detected by biochemical methods [31]. To determine if unknown AMP inducing this response could be synthesized by *A. pisum*, we tested whether the genes induced in aphids are indeed induced by known AMPs and whether PhoP and PmrA are involved in this regulation. We analyzed the resistance to AMPs of *arnB* and *dltB* mutants and examined their role during aphid infection.

### Regulation of AMP Resistance Genes

Resistance to AMP has been extensively studied in *S. enterica*. We first looked for the presence and induction in *D. dadantii* of homologues of genes which have been shown to play a role in AMP resistance in *S. enterica*. The two component regulators PhoP-PhoQ and PmrA-PmrB are present in *D. dadantii* but PmrD, which links both systems and allows PmrA-regulated genes to be regulated by PhoP is absent. In addition to *pmrC* and to the *arnB* operon, the *eptB* gene, which codes for another phosphoethanolamine adding enzyme, is present and induced 6-fold (Table 1). The oxygenase encoding *lpxO* and the palmitoyl transferase encoding *pagP* are present in *D. dadantii* but are not induced in aphid and *pagL* is absent. Thus, most of the genes involved in LPS modification in response to AMP in *S. enterica* have a homologue in *D. dadantii* but all are not induced in an aphid infection.

**Figure 1:**
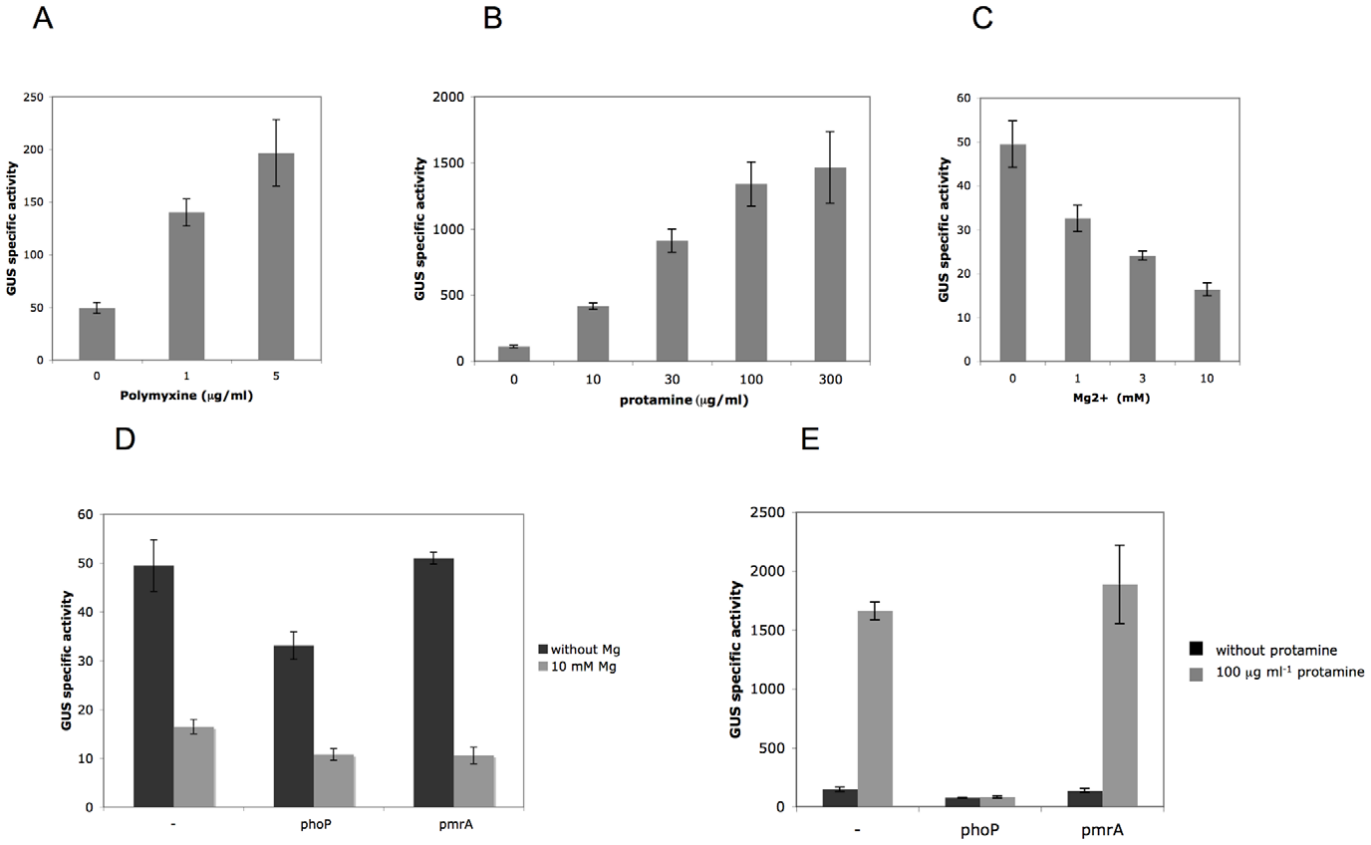
Regulation of *arnB*. The *arnB-uidA* fusion of strain A5256 was assayed in the presence of increasing concentrations of polymyxin (A), protamine (B), and Mg^2+^ (C). Effect of *phoP* and *pmrA* mutations on *arnB-uidA* regulation by Mg^2+^ (D) and protamine (E) was assayed. Cultures were performed in LB medium for A, C and D and in M63 medium for B and E since protamine precipitates in LB medium. Activities are the mean value from at least four separate experiments and are expressed in µmoles of *p*-nitrophenol produced per minute and per milligram of bacterial dry weight ± standard deviation.

Presence of AMP and growth in low Mg^2+^ medium are conditions inducing the PhoP-regulated genes in *S. enterica* and *E. coli,* including *arnB-F.* Regulation of *D. dadantii arnB* in these conditions was analyzed using a GUS reporter fusion. We tested two commonly used AMPs, polymyxin B and protamine. *arnB* was induced in the presence of both compounds (Fig. 1A and 1B). LB is a medium of low Mg^2+^ concentration [32], [33]. Addition of a high Mg^2+^ concentration (10 mM) in this medium repressed *arnB* expression five-fold (Fig. 1C). This repression is much lower than that described in *S. enterica*. To test whether the effect of polymyxin, protamine and Mg^2+^ on *arnB* occurs through PhoP or PmrA, the *arnB-uidA* fusion was assayed in these backgrounds. While its expression was unchanged in the *pmrA* mutant, it was reduced by one third in the *phoP* mutant, indicating that PhoP is an activator of *arnB* (Fig. 1D and 1E). In both backgrounds, repression by 10 mM Mg^2+^ was conserved, showing that neither PhoP nor PmrA are involved in this regulation (Fig. 1D). While induction by polymyxin was conserved in the *phoP* and *pmrA* mutant (data not shown), induction by protamine was abolished in the *phoP* background (Fig. 1E). This result indicates that regulation of *arnB* by AMPs involves two systems. One of them, the PhoP-PhoQ system, is sensing presence of protamine but is blind to polymyxin.

### Some of the Genes Induced in Aphid are Induced by AMP

**Figure 2:**
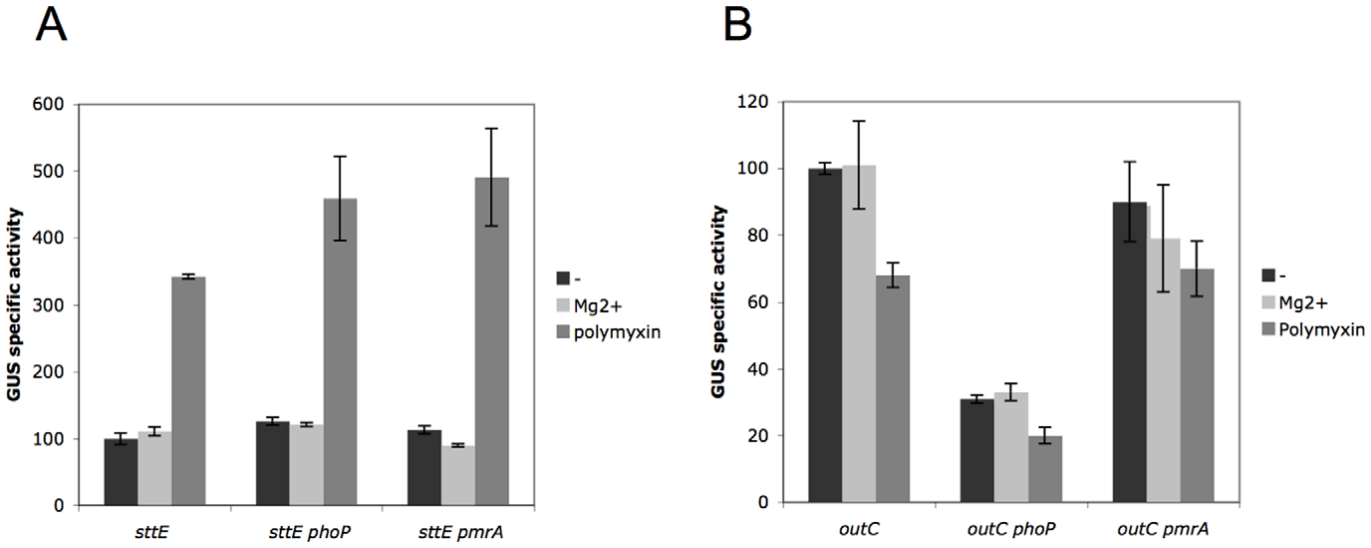
Regulation of *sstE* and *outC*. The *sttE-uidA* and *outC-uidA* fusions of strain A4206 and A1919, respectively, were assayed in the presence of 5 µg/ml of polymyxin or 10 mg/ml Mg^2+^ in the wt, *phoP* and *pmrA* backgrounds. Activities are the mean value from at least four separate experiments and are expressed in µmoles of *p*-nitrophenol produced per minute and per milligram of bacterial dry weight ± standard deviation.

To know whether induction or repression of genes observed when *D. dadantii* is infecting *A. pisum* could be due to the presence of AMPs, we analyzed the effect of protamine and polymyxin on the expression of four upregulated genes (*dltB*, *sotA*, *sotB*, and *sttE*) and of three downregulated ones (*outC*, *kdgM* and *kdgN*). None of these genes was differencially expressed in the presence of 100 µg ml^−1^ protamine (data not shown). In contrast, while the expression of *sotA*, *sotB*, *kdgM* and *kdgN* was not significantly affected by polymyxin, that of *outC* was reduced by 40% and that of *sttE* was increased by 3-fold, respectively, in the presence of that AMP (Fig. 2A and 2B, Table S3). Thus, not all genes induced in aphids are induced by polymyxin and a putative AMP produced by aphids could be a signal that modifies the expression of some of them.

The regulation of *sttE* and *outC* was studied in more details. Expression of none of these genes was controlled by Mg^2+^ concentration (Fig. 2A and 2B). Expression of *sttE* was not modified in a *phoP* or a *pmrA* background and in both cases polymyxin induction was conserved (Fig. 2A). The level of *outC* was identical in the *pmrA* background to that in the wt strain but was reduced three-fold in the *phoP* background, indicating that PhoP is an activator of *outC*. In both *phoP* and *pmrA* mutants, repression of *outC* by polymyxin was conserved (Fig. 2B). This confirms that PhoP and PmrA do not respond to polymyxin and that a gene regulated by PhoP is still sensitive to polymyxin in the absence of PhoP. In summary, regulation of genes involved in response to AMP is very different in *D. dadantii* from that described in *S. enterica* since neither polymyxin nor Mg^2+^ sensing occur through PhoP and PmrA and at least two regulators respond to different AMPs.

### *arnB* and *dltB* are Involved in Resistance to AMP

**Figure 3:**
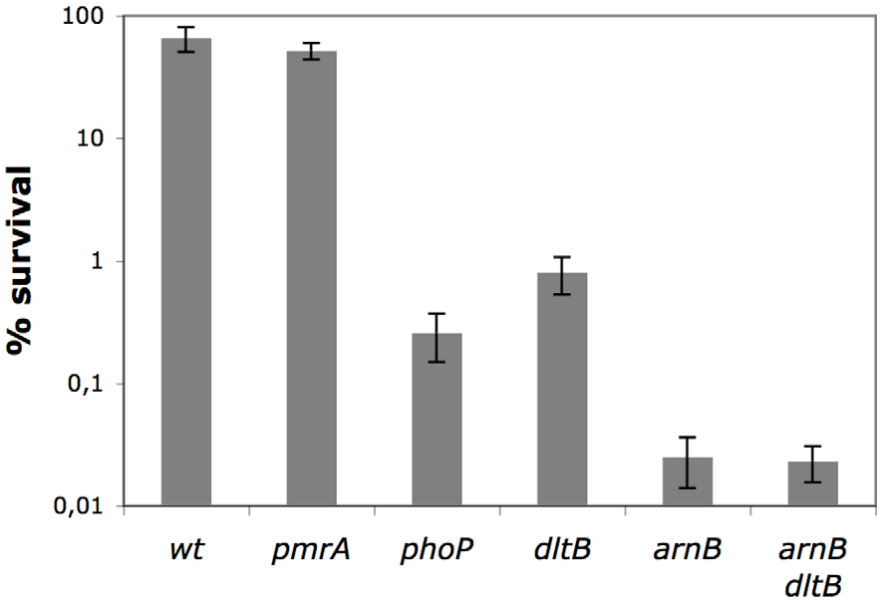
Survival of various mutants to polymyxin. Wild type and various mutants in genes involved in resistance to AMP (*phoP*, *pmrA*, *dltB*, *arnB*) were incubated in the presence of 1 µg ml^−1^ polymyxin for 1 h. Samples were diluted and plated on LB agar plates to assess bacterial viability. Survival values are relative to the original inoculum. Data correspond to mean values of three independent experiments.

To investigate whether *arnB*, *dltB*, *phoP* and *pmrA* are involved in resistance to AMP, a survival test to exposure to 1 µg ml^−1^ polymyxin was performed. The *pmrA* mutant was almost as resistant as the wt strain (Fig. 3). Thus PmrA-PmrB seems to play a limited role in resistance to AMP in *D. dadantii*. The *phoP* mutant showed more than 99% mortality after one hour exposure to polymyxin, showing that although PhoQ does not respond to that AMP, the PhoP-PhoQ system is involved in the regulation of genes required to resist to it. The *dltB* mutation had a strong effect on survival of the bacteria, leading to a 99% mortality. The most dramatic effect was observed with the *arnB* mutant with less than 0.1% of surviving bacteria after one hour (Fig. 3). This shows that LPS modification by the products of the *arnBCADTEF* operon is a main element of the resistance of *D. dadantii* to polymyxin and that absence of this modification leads to an increased bacterial susceptibility to this type of compounds. However, the *dltB* mutant survival was decreased, showing that, although its effect is limited when modification of LPS by 4-aminoarabinose can occur, alanylation of an unknown substrate by the *dlt* gene products is an important factor of resistance to AMP. No significant variation in the survival rate in the presence of polymyxin was observed when bacteria were grown in a LB medium containing 10 mM Mg^2+^ (data not shown).

**Figure 4:**
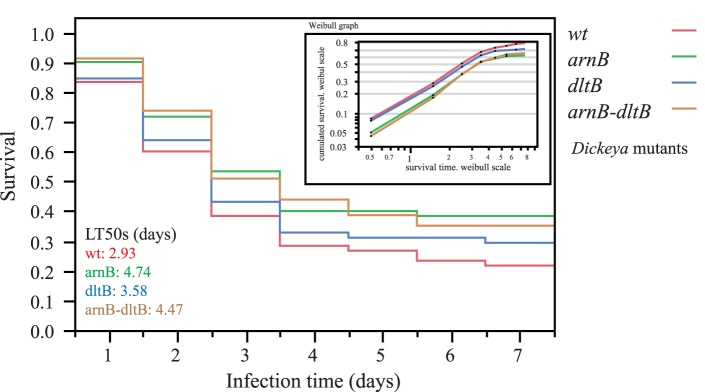
Survival of pea aphids after oral infection by wt and mutants of *D. dadantii* 3937. Survival is shown for aphids treated with wt bacteria (red), *arnB* (green), *dltB* (blue), and *dltB arnB* (brown) mutants. Results were obtained with 2×30 third instar aphid nymphs per treatment, including a diet-treated control (no mortality, not shown). The experiment was repeated twice with very similar results (p<0.06 in all comparisons of wt with *arnB* mutants). Median survival times (LT50s) were calculated with a Weibull fit (inlet), and give the following series [95% confidence intervals]: wt, 2.92 [2.18–3.92]; *arnB*, 4.74 [3.42–6.55]; *dltB* 3.58 [2.62–4.88] and *arnB*-*dltB* double mutant, 4.46 [3.23–6.18].

The *arnB*, the *dltB* and the *arnB dltB* mutants were then tested for their virulence in aphids. A comparison of the LT50s showed a slower mortality of aphids was observed with the *arnB* mutants but not with the *dltB* mutants (*P* = 0.042 for the wt versus *arnB* and *P* = 0.0671 for the wt versus *arnB dltB* double mutant comparisons, Log-rank non-parametric survival test), indicating a decreased ability of the *arnB* mutants to develop into the insect (Fig. 4). Thus, modification of LPS by the *arn* gene products is required for full virulence of the bacteria probably because it is involved in the resistance to AMPs produced by aphids.

### Inducible Peptides in Infected Pea Aphid

To try to identify the aphid AMPs, extracts from whole aphids, digestive tracts and hemolymph from control and *Dickeya*-challenged insects were separated by chromatography. All extracts gave very similar chromatographic traces at 280 nm (not shown) or 214 nm (Table S4 and Fig. S2). MALDI-ToF analysis of gut and haemolymph extract fractions did not show peptide peaks above 4500 Da, and few of them did differentiate the *Dickeya*-challenged modality (Table S4). Therefore, we focused our LC-MS analysis on the only samples that did show reproducible induced peaks in the bacteria-challenged samples (Figure S2), the digestive tract HPLC fractions (Table S4). Only one small peptide differentially induced in the *Dickeya*-challenged gut was found, namely the 13 amino acid C-terminal peptide from the ACYPI003154 gene product, a 14-3-3 family protein with conserved protein-protein interacting domains. This peptide, QDNDEPQEATGDN, has a net negative charge of -5 and could be an anionic AMP. This peptide was synthesized and its activity was tested against various bacteria. It had no antimicrobial activity against *D. dadantii*, *E. coli* and *Bacillus subtilis*. It was neither able to induce the expression of a *D. dadantii arnB-uidA* or *dltB-uidA* fusion (data not shown).

## Discussion

*D. dadantii* has the abilities to be both a plant and an insect pathogen. While the mechanisms of virulence towards plants are now well understood, factors required for the development of bacteria in aphids and to kill them are unknown. Cyt toxins seem to play only an accessory role in this process since a mutant devoid of toxin genes can still kill aphids. An analysis of the transcriptome of *D. dadantii* infecting aphids was performed to better understand the mechanisms of pathogenicity of bacteria towards insects. This technique was preferred to IVET or TraSH techniques used in other studies since it allows a global analysis of the genes induced and repressed which is not restricted to virulence factors required for survival in insects and allows to detect redundant factors that could be missed by other methods [21], [22]. The results showed a very important modification of the pattern of gene expression. However, no characterized toxin gene was found among the induced genes. New putative toxins are perhaps to be identified among the induced proteins of unknown function. Other factors required for pathogenesis may not have been detected because they are expressed early in the infection. Another possibility is that no additional toxin is required for *D. dadantii* to kill aphids or that toxins are expressed only during the early stage of infection. The high number of bacteria found in all the insect organs could be sufficient to provoke the death by bacteremia, as observed for *Pseudomonas aeruginosa* PA14 infecting *D. melanogaster* [34]. Genes induced in aphid body would just favor multiplication of the bacteria.

Insects are used as alternative models to identify virulence factors required for infection of more complex organisms, plant or animals, since a factor is often active in different models [22], [35], [36]. *D. dadantii* pathogenicity towards insect and plant seems to require a totally different set of genes since all the known plant virulence factors are strongly repressed or at least not affected in *A. pisum*. This opposition had already been observed for *cyt* gene regulation [19]. This reinforces the hypothesis that this bacterium may be faced with these two types of ecosystems and it has adaptive mechanisms to cope with a rapidly changing environment (e.g. ingestion by an insect). Such a dramatic change in gene expression profile has been observed for *Candidatus* Phytoplasma asteris OY-M grown *in planta* or in its insect host [37].

The transcriptome also reflects the response of the bacteria to the insect innate immune system. It consists of encapsulation, phagocytosis, melanisation and antimicrobial peptides and lysozyme synthesis [38]. For the two first factors, no known specific bacterial response has been identified. Melanisation catalyzed by the polyphenoloxidases produces reactive oxygene species which damage DNA. Bacteria respond to this stress by destroying reactive oxygene and reparing DNA. In *F. novicida* infecting *D. melanogaster* a large set of DNA repair and detoxification genes are induced [22]. In our experiment a very limited number of genes potentially involved in these processes are overexpressed (*mutM*, *iscR*, *rsxA*, *rsxB*), which could indicate that oxidative stress felt by bacteria is low. The low level of polyphenoloxidase activity measured in aphids with systemic infection by bacteria confirms that this pathway is of limited importance to fight bacterial infections [38].

Among the most induced genes, a number are involved in efflux and in a typical response to AMP. This let suppose that synthesis of this type of molecules could be the main response of *A. pisum* to bacterial infection. Whether *A. pisum* does produce AMP was up to date unknown, in spite of explicit searches. Genes coding for classical insect AMP were not found in the *A. pisum* genome and biochemical tests to identify them were unsuccessful [31], [38], [39]. However, induction of genes involved in AMP resistance typical of Gram negative (*arnB*) and Gram positive (*dltB*) bacteria strongly suggests that aphids do produce AMP. The reduced virulence to aphids of the *arnB* mutant probably results from its increased sensitivity to AMPs. Efflux transporters expel from the bacteria all kind of toxic molecules. Interestingly, the mutant in one of the efflux system induced in this study, GenID 15786-7-8, is more sensitive to protamine. Given the broad spectrum of this type of exporters, involvement of some of them in AMP efflux is possible.

AMPs are very diverse in sequence and structure but most of them are positively charged, allowing their interaction with the bacterial envelope. Modifications of LPS to mask the negative charges that allow interaction with AMP are one of the main responses to these compounds in many Gram negative bacteria. This response is generally induced by growth in Mg^2+^-limiting conditions or in the presence of AMP. Sensing these conditions occurs most often through PhoP-PhoQ and PmrA-PmrB. The wiring between the signals (Mg^2+^ and AMP), the regulators (PhoP and PmrA) and their targets may vary from species to species [32]; [10]. Our data show that the sensing capacities of PhoP-PhoQ and the PhoP-regulated genes in *D. dadantii* are different from what has been previously described in other bacteria. Growth in low Mg^2+^-medium is an inducing condition for the LPS-modifying gene *arnB*. However induction is low and is independent of the regulation of *arnB* by PhoP (Fig. 1C). The induction observed suggests that another Mg^2+^ sensing regulator controls *arnB* expression (Fig. 1C). Presence of an acidic patch in the sensor PhoQ is required for sensing Mg^2+^ in *S. enterica* and *E. tarda* [11], [40]. This acidic patch is not conserved in *D. dadantii* (SSEDKPT versus EDDDDAE in *S. enterica* PhoQ) which explains the blindness of PhoQ to Mg^2+^. A similar blindness of PhoQ due to an absence of acidic patch has been shown in *S. glossinidius*, an endosymbiont of the tse-tse fly [13]. This has been correlated to the adaptation of the bacteria to a steady environment where Mg^2+^ sensing is no longer required. A low Mg^2+^ concentration is supposed to mimic the conditions encountered in phagosomes by intracellular pathogens. As a plant pathogen, it is always found outside cells where divalent cation concentration (Mg^2+^, Ca^2+^) is high. Thus, a link between low Mg^2+^ and response to AMP may not be required for this bacteria.

A recent transcriptome analysis of *D. dadantii* treated with thionin, a plant AMP, identified 36 induced genes, most of them being regulated by *phoP* [15]. Very few of them are common with those upregulated in our analysis: the *arnB* operon genes, the *vfmE* regulator and an ABC tranporter (GenID 18174 and 18175). Thus, *D. dadantii* PhoP-PhoQ is able to sense certain AMPs since induction of *arnB* expression by protamine and thionine is abolished in a *phoP* mutant (Fig. 1E and [15]). However, all AMPs are not sensed by PhoP-PhoQ since induction by polymyxin of *arnB* and *outC* (Fig. 2 A and 2B) is conserved in a *phoP* background. This suggests existence of another AMP sensing system. In *P. aeruginosa*, *arnB*, which is induced by polymyxin, is not regulated by PhoP-PhoQ but by an other two-component system ParR-ParS [41], that has no homologue in *D. dadantii*. Polymyxin is inducing or repressing several genes that are induced or repressed in our transcriptomic analysis (*dltB*, *sttE*, *outC*). They could all be regulated by a polymyxin-sensing regulator that could also sense a yet unknown AMP synthesized by aphids.

*A. pisum* AMPs could not be identified by homology-based methods, indicating that they are probably limited in number and under rapid diversifying evolution. In our attempts to identify such an AMP by a biological method, we unambiguously characterized only one small peptide that was differentially present in bacteria-infected tissue extracts. This peptide is the C-terminal end of a 14-3-3 protein, a member of a well-known family of protein-protein interactants, with recent reported involvement in insect immunity, including control of AMP secretion [42], [43]. Although this peptide has the characteristics of an anionic AMP, it has no activity on *D. dadantii* or other tested bacteria. The mode of action of this type of AMPs is less known [44] but it could be active on *Buchnera aphidicola*, the aphid endosymbiont, which has an atypical membrane that does not contain LPS [45]. Alternatively, this peptide might be part of a signal transduction pathway of infected aphid gut, and thus falls outside the scope of the present study.

Analysis of induced genes allowed to detect other signals sensed by the bacteria when they infect *A. pisum*. In contrast to *E. coli* or *S. enterica*, PmrA does not control the *D. dadantii arnB* operon. *pmrA* inactivation modifies only weakly the resistance of the bacteria to polymyxin. However, it activates some genes which are induced in aphids but not by polymyxin and it allows their induction by Fe^3+^ (data not shown). Thus, in *D. dadantii*, *pmrA* does not seem involved in the response to AMP and it is not known if Fe^3+^ is the only signal it can sense. Induction of the synthesis of the nitrate and nitrite dehydrogenases (NarI and NirB) probably results from anaerobic conditions in some aphid compartments. Our work allowed for the first time to decipher the global gene expression of a bacterium during an insect infection and showed that induction of genes in insects could be the result of many different signals, including several AMPs, the PmrA signal, anaerobiosis, sensed by a complex set of regulators.

## Methods

### Bacterial Strains and Growth Conditions

Bacterial strains, phages, plasmids and oligonucleotides used in this study are described in Table S5. *D. dadantii* and *E. coli* cells were grown at 28 and 37°C respectively unless otherwise stated in LB medium or M63 minimal medium supplemented with a carbon source (0.2%, w/v). When required antibiotics were added at the following concentration: ampicillin, 100 mg l^−1^, kanamycin and chloramphenicol, 25 mg l^−1^, tetracycline, 20 mg l^−1^. For aphid ingestion tests, bacteria were resuspended in AP3 medium [46]. Media were solidified with 1.5% (w/v) Difco agar. All mutations were in the wt background for insect tests and in the A350 background for enzymatic assays. Transduction with phage ΦEC2 was performed according to Résibois *et al.* [47].

### Strain Construction

To construct strain A5256 that contains an *arnB-uidA* fusion, a 1.8 kb DNA fragment containing *arnB* was amplified with primers arnB+ (ggatggatgaatgtttgcggctg) and arnB- (cgcggccgaattgtcgctgctg). The resulting DNA fragment was inserted into the pGEM-T plasmid (Promega). A *uidA*-kanR cassette was prepared from plasmid pUIDK1 [48] by digestion with EcoRI and inserted into the unique MunI site of *arnB*. To construct strain A5248 that contains a *pmrA*-Cm^R^ insertion, a 1.9 kb DNA fragment containing *pmrA* was amplified with primers pmrA+ (ggaatatcaggcgccgcttg) and pmrA- (atatggtgtatgcccggcgg). The resulting DNA fragment was inserted into the pGEM-T plasmid (Promega). A Ω-Cm^R^ cassette was prepared from plasmid pHP45-ΩCm [49] by digestion with BamHI and inserted between the two BamHI sites of *pmr*A. To construct strain A5394 and A5399 that contain a *dltB-uidA* fusion and a *dltB*-Cm^R^ insertion respectively, a 1.7 kb DNA fragment containing *dltB* was amplified with primers dltB+ (aaagtgctgcgacattctgg) and dltB- (cgcgggcttgagtaatgccg). The resulting DNA fragment was inserted into the pGEM-T plasmid (Promega). A *uidA*-kanR cassette was prepared from plasmid pUIDK3 by digestion with XmaI and inserted into the unique BspEI site of *dltB*. A Cm^R^ cassette was prepared from plasmid CKC15 [4] by digestion with *Xma*I and inserted into the unique BspEI site of *dltB*. All the resulting constructs were inserted into the *D. dadantii* chromosome by recombination in low phosphate medium [50]. Recombinations were checked by PCR.

### Aphid Strains and Infection Experiments

The aphid clone used was LL01, an alfalfa collected clone long-established in the lab and grown on broad beans (*Vicia faba* cv. Aquadulce). Inoculations by ingestion were performed as described in Grenier *et al.* [17]. Forty third instar aphid nymphs, fed on broad beans, were maintained for 24 h on an AP3 diet, containing bacteria at 10^6^ bacteria ml^−1^ before being placed back onto bean leaves at 20°C ( =  day 1). To evaluate the survival rate of infected pea aphids the number of survivors was counted every day for 7 days. For each strain, two independent biological replicates were tested.

### RNA Preparation

To extract RNA from *D. dadantii* 3937 grown AP3 medium, bacteria grown for 24 h (OD_600_>2) were harvested by centrifugation and total RNA were extracted using TRIzol® reagent (Invitrogen) according to the manufacturer recommendations. To extract *D. dadantii* 3937 RNA from infected aphids, bacterial inoculation by ingestion was performed as described in [17]. For each assay, 40 third instar aphid nymphs, fed on broad beans, were maintained for 24 h on an AP3 diet containing 10^6^ bacteria ml^−1^, before being placed back onto the beans at 20°C. After 3 days of infection aphids were crushed in two microcentrifuge tubes (2×20 aphids), with a sterilized pestle, in 250 µl of RNAprotect Bacteria Reagent (Qiagen). 250 µl of cold DEPC-treated water were then added. The suspension (≈ 1 ml) was centrifuged at 10000 g for 10 min at 4°C on a discontinuous 10% to 50% (w/w) sucrose gradient in Tris-HCl 10 mM pH 8.0, 1 mM EDTA in order to eliminate mitochondria, host nuclei and cellular fragments. Bacterial cells, localized in a green band, were collected with a glass pipette (V ≈ 1.5 ml) and pooled. Total RNA were then extracted using TRIzol® reagent (Invitrogen) following the manufacturer recommendations.

Bacterial RNA from all samples was treated with DNase I of the TURBO DNAfree™ kit (Ambion) following the manufacturer instructions. Absence of genomic DNA contamination was checked by PCR with two primer pairs cytA-up - cytB-down and cytC-up - *cytD*-down [19]. Purified RNA was quantified on the basis of its absorption at 260 nm using an ND 100 Nanodrop spectrophotometer and visualized on an agarose gel to check quality. Enrichment for mRNA was required to increase the signal intensity on the microarray and reach the NimbleGen® sample requirements. A step of RNA amplification was performed with the MicrobEnrich™ kit (Ambion) following the manufacturer recommendations. In order to ensure that this additional step had no influence on the relative gene expression pattern, a part of purified RNA from all samples was stored at −80°C for later confirmation by RT-qPCR. Amplified RNA was finally quantified using Nanodrop spectrophotometer and visualized on an agarose gel to check quality before being sent to NimbleGen for hybridization on *D. dadantii* microarrays.

### Microarray Design

The microarrays used in this study were custom designed and produced by NimbleGen Systems, Inc. (Madison, WI), based on the annotated sequence (version number 6) of *D. dadantii* (available at https://asap.ahabs.wisc.edu/asap/logon.php), which comprised 4753 coding sequences (CDS). The microarrays consisted of 70-mer oligonucleotides with 5 perfect match probes per CDS, in three blocks on the array. For microarray analyses, cDNA was synthesized, labeled, and hybridized by NimbleGen Systems, Inc. For each condition, three independent biological replicates were tested. The experimental design of array, and all raw and processed data, were deposited as a GEO archive (accession number GSE42585).

### Data Analysis and Statistical Procedures

All statistical tests, including aphid survival analyses (non parametric and parametric models, of which a Weibull adjustment was found to best describe the insect survival trend), were performed with JMP software (V 9.0.3, SAS Inc Cary 27513-2414 USA). Aphid EST analysis was performed with the MacVector software, including the assembler module (V 12.6, MacVector Cary NC 27519 USA). The two aphid gut libraries ID0AFF (control) and ID0AAG (Dickeya-treated) [51] were assembled altogether with MacVector, blasted against V2.1 *A. pisum* genome assembly (mRNAs), and all hits were discarded, in order to retrieve all small and non-conventional gene models not present in the V2.1 genome assembly. The 527 contigs retrieved were then filtered-off for contaminating *Buchnera* proteins, translated and the resulting partial ORFs used for additional target analysis in the LC MS-MS analyses. Micro-arrays were analyzed using the expression module from the CLC-Bio Main Workbench V6 package. Quality control was checked by data analysis and principal component and group cluster analysis, which easily grouped biological conditions, biological replicates and technical replicates in this order. Normalized data (scaling, median) were analyzed by K-means hierarchical clustering of genes (5 clusters) and by gene set enrichment analysis, GSEA [52] on GO classifications.

### Quantitative Real-time PCR Control

After microarray data results, qRT-PCR were performed on several genes to confirm the relative gene expression pattern. Same RNA samples used for RNA amplification and next array hybridization were used for these experiments. RT was performed using SuperScript II reverse transcriptase (Invitrogen) with 500 ng of total RNA and 25 ng of random hexamer primers, according to the manufacturer’s protocol (first strand cDNA synthesis). One microliter of the RT reaction mixture was added as a template to the Qbiogen Sybr Green mix for PCR with gene-specific primers. Primers used in this work are listed in Table S6. The thermal cycling reactions were performed using a LightCycler (Roche) according to the following conditions: an initial step at 95°C for 10 min, followed by 40 cycles at 95°C for 15 s, 55°C for 15 s, and 72°C for 20 s. Two housekeeping genes, *rpoA* and *ffh*, were used as standards to obtain normalized target gene expression ratios as described in a previous study of *D. dadantii* transcriptome [53]. The qPCR efficiency was 1.80 and 1.96 for *rpoA* and *ffh*, respectively. The statistical program used to analyze the data was the Relative Expression Software Tool (REST 2009 V2.0.13) [54]. The specificity of the PCR primers was verified with a melting curve analysis using the LightCycler® 480 Software (Roche).

### Peptide Extraction and Chromatographic Analysis

Aphids were extracted as young day 2–5 parthenogenetic females, using either a flash-hemolymph extraction technique [31], or a gut dissection following the same TFA 0.1% extraction procedure [51]. Phenyl-thiourea was used for SDS-PAGE hemolymph purity controls, but omitted when HPLC was used subsequently. Aphid acid extracts (whole-body WB, haemolymph HY or digestive tract DT) were then separated using a Dionex UPLC System Ultimate 3000 and a Waters XBridge BEH130 C18 column (2.1×150 mm, 5 µm particle size, 130-Å porosity) at 40°C, with a precolumn. Absorbance was monitored at 214 and 280 nm. The solvent system was 0.1% TFA in water (Solvent A) and 0.09% TFA in 70% acetonitrile/water (Solvent B) with a flow rate of 0.4 ml min^−1^ and the gradient is shown on figures. Fractions were collected every 30 sec in ELISA plates and dried using a Speed-Vac concentrator.

### Peptide Identification by Mass Spectrometry

Plate-collected UPLC fractions were dissolved in 10 µl H_2_O/acetonitrile 99/1 acidified by 0.1% of TFA. The analysis was performed on a nanoACQUITY Ultra-Performance-LC (UPLC, Waters, Milford, MA). 4µL of each sample were loaded on a 20×0.18 mm, 5 µm Symmetry C18 precolumn (Waters Corp.), and the peptides were separated on a ACQUITY UPLC® BEH130 C18 column (Waters Corp.), 75 µm×200 mm, 1.7 µm particle size. The solvent system consisted of 0.1% formic acid in water (solvent A) and 0.1% formic acid in acetonitrile (solvent B). Trapping was performed during 3 min at 5 µL min^−1^ with 99% of solvent A and 1% of solvent B. Elution was performed at a flow rate of 300 nL min^−1^, using 1–40% gradient (solvent B) over 35 min at 50°C followed by 65% (solvent B) over 5 min. The MS and MS/MS analyzes were performed on the SYNAPT™, an hybrid quadrupole orthogonal acceleration time-of-flight tandem mass spectrometer (Waters, Milford, MA) equipped with a Z-spray ion source and a lock mass system. The capillary voltage was set at 3.5 kV and the cone voltage at 35 V. Mass calibration of the TOF was achieved using phosphoric acid (H_3_PO_4_) on the [50;1800] m/z range in positive mode. Online correction of this calibration was achieved using lock-mass on product ions derived from the [Glu1]-fibrinopeptide B (GFP). The ion (M+2H)2+ at m/z 785.8426 is used to calibrate MS data and the fragment ion (M+H)+ at m/z 684.3469 is used to calibrate MS/MS data during the analysis. For tandem MS experiments, the system was operated with automatic switching between MS and MS/MS modes (MS 0.5 s/scan on m/z range [250;1500] and MS/MS 0.7 s/scan on m/z range [50;1800]). The 3 most abundant peptides (intensity threshold 60 count s^−1^), preferably doubly and triply charged ions, were selected on each MS spectrum for further isolation and CID fragmentation. Fragmentation was performed using argon as the collision gas. The complete system was fully controlled by MassLynx 4.1 (SCN 712, Waters, Milford, MA). Raw data collected during nanoLC-MS/MS analyses were processed and converted with ProteinLynx Browser 2.4 (Waters, Milford, MA) into.pkl peak list format. Normal background substraction type was used for both MS and MS/MS with 5% threshold and polynomial correction of order 5, and deisotoping was performed. The MS/MS data were analyzed using the MASCOT 2.4 algorithm (Matrix Science) to search against the aphid proteome database (v 2.1), concatenated with additional translation of normal/infected digestive tract EST contigs [51], locally assembled with Vector 12.6 (Mac Vector Inc. Cary NC 27519 USA).

### Enzymatic Assays

β-glucuronidase assays were performed on toluenized extracts of cells grown to exponential phase by the method of Bardonnet *et al.* [48] using *p*-nitrophenyl-β-D-glucuronate as substrate.

### Antimicrobial Peptide Resistance Assays

Stationary phase grown cultures of *D. dadantii* strains were diluted 10^3^-fold in LB medium. 1 ml of dilution was placed in an 1.5 ml Eppendorf tube and AMP was added. After 1 hour of incubation at room temperature, bacteria were diluted and plated on LB plates. Colonies were counted after 2 days of growth at 28°C.

## Supporting Information

Figure S1 **Validation of microarray results by qRT-PCR.** (A) Expression ratios of *D. dadantii* genes during aphid infection versus growth in liquid medium measured by qRT-PCR. Expression of each gene was normalized to the expression of the two housekeeping genes *rpoA* and *ffh*. A positive expression ratio indicates upregulated genes during aphid infection, and a negative expression ratio indicates downregulated genes during aphid infection. Standard error ranges were calculated from the data from three independent biological replicates. (B) Comparison of gene expression measurements by microarray approach and real-time qRT PCR. The correlation coefficient (R^2^) is given.
(DOC)

Figure S2 **HPLC traces at 214 nm of haemolymph and digestive tract acidic extracts from the pea aphid challenged or not by** ***Dickeya dadantii*** **.** The horizontal bar shows the collected (differential) fractions.
(EPS)

Table S1 **GSEA analysis of Gene Ontology categories in insect-infecting bacteria** ***vs*** **control** ***Dickeya dadantii*** **cells.**
(DOCX)

Table S2 ***D. dadantii*** **genes induced or repressed in aphid with a P value <0.002.**
(XLSX)

Table S3 **Expression of selected genes in the presence of polymyxin.**
(DOC)

Table S4 **Summary of peptide LC MS analysis.**
(DOCX)

Table S5 **Bacterial strains used in this study.**
(DOC)

Table S6 **Oligonucleotides used for RT-qPCR experiments.**
(DOC)
