# Supplementary material for: Transcriptome of Dickeya dadantii Infecting Acyrthosiphon pisum Reveals a Strong Defense against Antimicrobial Peptides
Source: PLoS One. 2013 Jan 14;8(1):e54118. doi: 10.1371/journal.pone.0054118 (PMC3544676; doi:10.1371/journal.pone.0054118)
Supplement: Figure S1 — Validation of microarray results by qRT-PCR. (A) Expression ratios of D. dadantii genes during aphid infection versus growth in liquid medium measured by qRT-PCR. Expression of each gene was normalized to the expression of the two housekeeping genes rpoA and ffh. A positive expression ratio indicates upregulated genes during aphid infection, and a negative expression ratio indicates downregulated genes during aphid infection. Standard error ranges were calculated from the data from three independent biological replicates. (B) Comparison of gene expression measurements by microarray approach and real-time qRT PCR. The correlation coefficient (R2) is given. (DOC) [file pone.0054118.s001.doc]

A.

| **Gene** | **qPCR efficiency** | **Expression ratio** | **Standard error range** | ***P value*** |
| --- | --- | --- | --- | --- |
| GenID 19611 | 1.81 | 172.1 | 28.89 – 515.74 | 0.009 |
| *arnB* | 1.84 | 116.5 | 40.56 – 254.97 | 0.027 |
| GenID15786 | 1.97 | 82.3 | 63.22 – 116.98 | 0.060 |
| *sotA* | 1.87 | 54.7 | 32.14 – 91.70 | 0.019 |
| *pmrC* | 1.90 | 42.6 | 32.66 – 62.56 | 0.019 |
| *sotB* | 1.81 | 39.8 | 25.00 – 79.44 | 0.065 |
| *pelE* | 1.96 | -76,9 | -100.00 – -47.62 | 0.033 |
| *kdgM* | 1.99 | -142.9 | -250.00 – -90.90 | 0.033 |
| *kdgN* | 1.94 | -166.7 | -333.33 – -111.11 | 0.042 |

**B.**


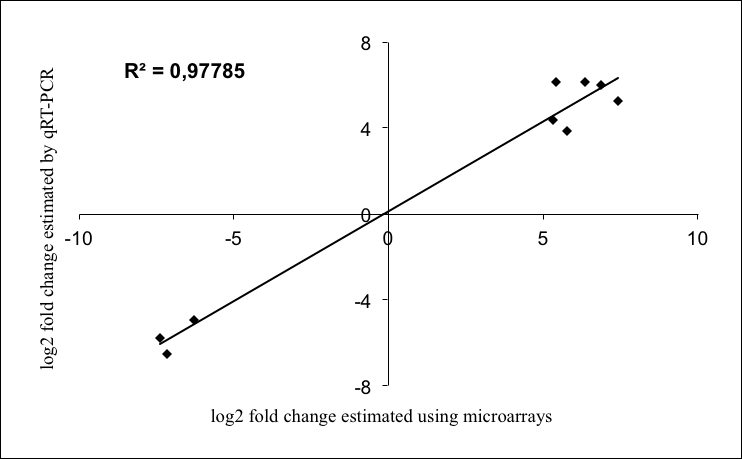


**Figure S1. Validation of microarray results by qRT-PCR.** (A) Expression ratios of *D. dadantii* genes during aphid infection versus growth in liquid medium measured by qRT-PCR. Expression of each gene was normalized to the expression of the two housekeeping genes *rpoA* and *ffh*. A positive expression ratio indicates upregulated genes during aphid infection, and a negative expression ratio indicates downregulated genes during aphid infection. Standard error ranges were calculated from the data from three independent biological replicates. (B) Comparison of gene expression measurements by microarray approach and real-time qRT PCR. The correlation coefficient (R^2^) is given.
