# Supplementary material for: Transcriptome of Dickeya dadantii Infecting Acyrthosiphon pisum Reveals a Strong Defense against Antimicrobial Peptides
Source: PLoS One. 2013 Jan 14;8(1):e54118. doi: 10.1371/journal.pone.0054118 (PMC3544676; doi:10.1371/journal.pone.0054118)
Supplement: Table S1 — GSEA analysis of Gene Ontology categories in insect-infecting bacteria vs control Dickeya dadantii cells. (DOCX) [file pone.0054118.s003.docx]

**Table S1**: GSEA analysis ^a^ of Gene Ontology categories in insect-infecting bacteria *vs* control *Dickeya dadantii* cells

| GO Category ^b^ | Category description | Cat. size | Statistic test ^c^ | Lower tail  (p-value) ^d^ | Upper tail (p-value) ^d^ |
| --- | --- | --- | --- | --- | --- |
| GO cc |  |  |  |  |  |
|  | membrane | 291 | -12,05 | **9,30E-3** | 0,99 |
|  | intracellular | 214 | -8,45 | **0,04** | 0,96 |
|  | outer membrane-bounded periplasmic space | 35 | 17,05 | 0,96 | **0,04** |
|  | ATP-binding cassette (ABC) transporter complex | 11 | 22,18 | 0,99 | **0,01** |
| GO mf |  |  |  |  |  |
|  | RNA binding | 67 | -34,59 | **0,00** | 1,00 |
|  | tRNA binding | 16 | -33,60 | **0,00** | 1,00 |
|  | structural constituent of ribosome | 20 | -19,41 | **8,00E-4** | 1,00 |
|  | nucleotide binding | 347 | 0,18 | **4,20E-3** | 1,00 |
|  | amino acid transmembrane transporter activity | 11 | -11,27 | **0,02** | 0,98 |
|  | kinase activity | 15 | 22,33 | 0,99 | **0,01** |
|  | sugar:hydrogen symporter activity | 14 | 30,12 | 1,00 | **1,10E-3** |
|  | catalytic activity | 439 | 55,58 | 1,00 | **0,00** |
| GO bp |  |  |  |  |  |
| GO:0006412 | translation | 90 | -51,42 | **0** | 1 |
| GO:0009306 | protein secretion | 25 | -16,72 | **7,00E-04** | 1 |
| GO:0007047 | cellular cell-wall organization | 14 | -17,57 | **1,50E-03** | 1 |
| GO:0006364 | rRNA processing | 17 | -16,02 | **2,50E-03** | 1 |
| GO:0006396 | RNA processing | 13 | -15,3 | **4,60E-03** | 1 |
| GO:0008652 | cellular amino acid biosynthetic process | 16 | -13,03 | **9,00E-03** | 0,99 |
| GO:0001522 | pseudouridine synthesis | 10 | -12,32 | **0,01** | 0,99 |
| GO:0009058 | biosynthetic process | 31 | -7,66 | **0,04** | 0,96 |
| GO:0008610 | lipid biosynthetic process | 11 | -9,38 | **0,04** | 0,96 |
| GO:0006350 | DNA-dependent transcription | 202 | 26,26 | 0,96 | **0,04** |
| GO:0045454 | cell redox homeostasy | 16 | 21,9 | 0,98 | **0,02** |
| GO:0006950 | response to stress | 12 | 26,59 | 1 | **4,20E-03** |
| GO:0005975 | carbohydrate metabolic process | 88 | 40,69 | 1 | **1,00E-04** |
| GO:0008152 | metabolic process | 323 | 44,76 | 1 | **1,00E-04** |
| GO:0006099 | tri-carboxylic acid cycle | 13 | 48,2 | 1 | **0** |
| GO:0006096 | glycolysis | 10 | 38,41 | 1 | **0** |
|  |  |  |  |  |  |

a : Gene Set Enrichment Analysis, as implemented in CLC-Bio main workstation, according to Subramanian *et al.* (2005).

b : Gene Ontology classifications :
 cc : cell compartment, mf : molecular function, bp : biological process.

c : GSEA statistic test.

d : a small lower or upper tail p-value for an annotation category is an indication that genes in this category viewed as a whole are perturbed among the groups in the considered experiment; lower tails are underexpressed under infection and upper tails are overexpressed ; only p-values < 0.05 are listed.

Subramanian, A., Tamayo, P., Mootha, V.K., Mukherjee, S., Ebert, B.L., Gillette, M.A., *et al.* (2005) Gene set enrichment analysis: a knowledge-based approach for interpreting genome-wide expression profiles, PNAS. 102 :15545–15550.
