# Supplementary material for: Transcriptome of Dickeya dadantii Infecting Acyrthosiphon pisum Reveals a Strong Defense against Antimicrobial Peptides
Source: PLoS One. 2013 Jan 14;8(1):e54118. doi: 10.1371/journal.pone.0054118 (PMC3544676; doi:10.1371/journal.pone.0054118)
Supplement: Table S3 — Expression of selected genes in the presence of polymyxin. (DOC) [file pone.0054118.s005.doc]

Table S3 : Expression of selected genes in the presence of polymyxin.

|  | LB | LB + polymyxin |
| --- | --- | --- |
| *sotB* | 67 + 6 | 89 + 8 |
| *sotA* | 12.3 + 2.1 | 9.3 + 0.8 |
| *kdgM* | 1520 + 360 | 1230 + 140 |
| *kdgN* | 1810 + 360 | 1640 + 390 |

Strains containing a fusion of the indicated gene with GUS were grown in LB medium in the presence or absence of 5 μg ml^-1^ polymyxin. β-glucuronidase activity was measured in four independant cultures and the mean value + SD in given.
