# Supplementary material for: Transcriptome of Dickeya dadantii Infecting Acyrthosiphon pisum Reveals a Strong Defense against Antimicrobial Peptides
Source: PLoS One. 2013 Jan 14;8(1):e54118. doi: 10.1371/journal.pone.0054118 (PMC3544676; doi:10.1371/journal.pone.0054118)
Supplement: Table S4 — Summary of peptide LC MS analysis. (DOCX) [file pone.0054118.s006.docx]

Table S4 : Summary of peptide LC MS analysis.  Differential hits from *Dickeya*-challenged samples identified in LC or MS experiments.

|  | Whole aphid WB | Haemolymph HY | Gut DT |
| --- | --- | --- | --- |
| HPLC separation | Complex  poorly resolved chromatogram | Resolved  no-differential chromatogram | Resolved  minor differential chromatogam |
| Differential Maldi-ToF hit on HPLC fractions | ND | Yes, minor | Yes |
| masses (m/z MH+) of differential hits |  | - | 931.382 974.550 1433.814 1277.767 1635.947 1509.813 |
| MS-MS hits (ACYPIs ^a^) | ND | ND | Yes |
| non differential (100% identity) |  |  | ACYPI010015 (14AA) ACYPI005624 (15AA)  ACYPI019765 (13AA)  ACYPI000474 (13AA)  ACYPI080190 (13AA)  ACYPI010127 (11AA)  ACYPI009765 (11AA) |
| Differential |  |  | **ACYPI003154** (14AA)  14-3-3 protein theta |

ND: not determined; see supplementary Fig S2 for sample chromatograms.

a: ACYPIs (V2.1) are the official IAGC identifiers for the pea aphid genes
