# Supplementary material for: Transcriptome of Dickeya dadantii Infecting Acyrthosiphon pisum Reveals a Strong Defense against Antimicrobial Peptides
Source: PLoS One. 2013 Jan 14;8(1):e54118. doi: 10.1371/journal.pone.0054118 (PMC3544676; doi:10.1371/journal.pone.0054118)
Supplement: Table S5 — Bacterial strains used in this study. (DOC) [file pone.0054118.s007.doc]

Table S5 : bacterial strains used in this study

| Strain | Genotype | Reference |
| --- | --- | --- |
| 3937 | *D. dadantii* wild type | Laboratory collection |
| A350 | *rafR ganB* | Hugouvieux-Cotte-Pattat and Robert-Baudouy, 1985 |
| A1919 | *rafR ganB outC*::*uidA*-kan^R^ | Condemi*ne et a*l., 1992 |
| A3145 | *rafR ganB sotA*::*uidA*-kan^R^ | Condemine, 2000 |
| A3501 | *rafR ganB sotB*::*uidA*-kan^R^ | Condemine, 2000 |
| A3573 | *rafR ganB kdgM*::*uidA*-kan^R^ | Bl*ot et a*l., 2002 |
| A4194 | *rafR ganB phoP*::Cm^R^ | Laboratory collection |
| A4205 | *rafR ganB kdgN*::*uidA*-kan^R^ | Condemine and Ghazi, 2007 |
| A4206 | *rafR ganB sttE*::*uidA*-kan^R^ | Ferrandez and Condemine, 2008 |
| A5248 | *rafR ganB pmrA*::Cm^R^ | This work |
| A5256 | *rafR ganB arnB*::*uidA*-kan^R^ | This work |
| A5394 | *rafR ganB dltB*::*uidA*-kan^R^ | This work |

Blot, N., Berrier, C., Hugouvieux-Cotte-Pattat, N., Ghazi, A., and Condemine, G. (2002) The oligogalacturonate-specific porin KdgM of *Erwinia chrysanthemi* belongs to a new porin family. *J Biol Chem* **277**: 7936-7944.

Condemine, G., Dorel, C., Hugouvieux-Cotte-Pattat, N., and Robert-Baudouy, J. (1992) Some of the out genes involved in the secretion of pectate lyases in *Erwinia chrysanthemi* are regulated by *kdgR*. *Mol Microbiol* **6**: 3199-3211.

Condemine, G. (2000) Characterization of SotA and SotB, two *Erwinia chrysanthemi* proteins which modify isopropyl-beta-D-thiogalactopyranoside and lactose induction of the Escherichia coli lac promoter. *J Bacteriol* **182**: 1340-1345.

Condemine, G., and Ghazi, A. (2007) Differential regulation of two oligogalacturonate outer membrane channels, KdgN and KdgM, of *Dickeya dadantii* (*Erwinia chrysanthemi*). *J Bacteriol* **189**: 5955-5962.

Ferrandez, Y., and Condemine, G. (2008) Novel mechanism of outer membrane targeting of proteins in Gram-negative bacteria. *Mol Microbiol* **69**: 1349-1357.

Hugouvieux-Cotte-Pattat, N., and Robert-Baudouy, J. (1985) Lactose metabolism in *Erwinia chrysanthemi*. *J Bacteriol* **162**: 248-255.
