# Supplementary material for: Transcriptome of Dickeya dadantii Infecting Acyrthosiphon pisum Reveals a Strong Defense against Antimicrobial Peptides
Source: PLoS One. 2013 Jan 14;8(1):e54118. doi: 10.1371/journal.pone.0054118 (PMC3544676; doi:10.1371/journal.pone.0054118)
Supplement: Table S6 — Oligonucleotides used for RT-qPCR experiments. (DOC) [file pone.0054118.s008.doc]

Table S6: oligonucleotides used for RT-qPCR experiments

| **Gene** | **Primer name** | **Primer sequence (5’-3’)** | **qPCR efficiency** |
| --- | --- | --- | --- |
| *rpoA* | rpoA-F | AAACCGCGCCTGGTAGATA | 1.80 |
|  | rpoA-R | CCTTTCAGGTTGAGCAGGAT |  |
| *ffh* | ffh-F | TGGTGCGTGATTTCATCAAT | 1.96 |
|  | ffh-R | CTTACCAACGCTGGTGGTTT |  |
| *arnB* | arnB-F | CCGCGTTAGGTGAGGAAGA | 1.84 |
|  | arnB-R | AGGTGAGCGACGGTGTGAT |  |
| *sotA* | sotA-F | GCTCCCCACGCTCAGTCT | 1.87 |
|  | sotA-R | ATAGTTACGGTTCCAGGCATACA |  |
| *sotB* | sotB-F | CGTCGCCTGGAACTTTACC | 1.81 |
|  | sotB-R | GCCGAGATACTGACCGATGAC |  |
| *pelE* | pelE-F | GGCGACGTCAACCACAAA | 1.96 |
|  | pelE-R | CAAAGCCGCAACCATTCA |  |
| *pmrC* | pmrC-F | CGGCTACGATGAACAACTGG | 1.90 |
|  | pmrC-R | TTCGACGCCGCTAAACAAT |  |
| *kdgM* | kdgM-F | TTGGCTGTTGCATCTCTGG | 1.99 |
|  | kdgM-R | AACGTAGCTGGCGACAACTT |  |
| *kdgN* | kdgN-F | GATTTCGCACCGTTTTGAGA | 1.94 |
|  | kdgN-R | ATAAGGGCGATAGCTGTTGGTA |  |
| GenID 19611 | 19611-F | GCGCTGATTTGGGTGATAGT | 1.81 |
|  | 19611-R | CATTGACGCGCTGAGTGAC |  |
| GenID 15786 | 15786-F | ATTGGCGTGGCGTTTTTCT | 1.97 |
|  | 15786-R | CCGCCCATCTGATTGAGGTA |  |
